# MTHFD1 Regulates Autophagy to Promote Growth and Metastasis in Colorectal Cancer via the PI3K‐AKT–mTOR Signaling Pathway

**DOI:** 10.1002/cam4.70267

**Published:** 2024-11-21

**Authors:** Zhihao Li, Haoxian Ke, Jiawei Cai, Shubiao Ye, Junfeng Huang, Chi Zhang, Ming Yuan, Ping Lan, Xianrui Wu

**Affiliations:** ^1^ Department of General Surgery The Sixth Affiliated Hospital, Sun Yat‐sen University Guangzhou Guangdong China; ^2^ Department of General Surgery Guangdong Provincial Key Laboratory of Colorectal and Pelvic Floor Diseases, The Sixth Affiliated Hospital, Sun Yat‐sen University Guangzhou Guangdong China; ^3^ Department of Gastrointestinal Surgery Sun Yat‐Sen Memorial Hospital, Sun Yat‐sen University Guangzhou Guangdong China

**Keywords:** autophagy, colorectal cancer, invasion, migration, MTHFD1, PI3K‐AKT‐mTOR, proliferation

## Abstract

**Objectives:**

Methylenetetrahydrofolate dehydrogenase 1 (MTHFD1) is the enzyme with the activities of methylenetetrahydrofolate dehydrogenase, methylenetetrahydrofolate cyclohydrolase, and formyltetrahydrofolate synthetase. Our aim was to elucidate the function of MTHFD1 in colorectal cancer (CRC).

**Methods:**

In vitro assessments of the proliferation, invasion, and migration abilities of CRC cells were conducted using Immunohistochemistry, Transwell invasion assays, Western blot (WB), and Cell counting Kit‐8 assays. WB was also utilized to measure autophagy protein levels and PI3K‐AKT‐mTOR signaling pathway expression. Furthermore, the role of MTHFD1 was evaluated in vivo by using subcutaneous xenograft tumor models and lateral tail vein metastasis models of human CRC in nude mice.

**Results:**

Overexpression of MTHFD1 promoted the abilities of tumorigenesis and metastasis in CRC in vitro and in vivo and reduced autophagy, attributing to the PI3K‐AKT‐mTOR signaling pathway in CRC cells. In contrast, the down‐regulation of MTHFD1 increased autophagy and suppressed their proliferation, migration, and invasion.

**Conclusions:**

MTHFD1 can modulate the PI3K‐AKT‐mTOR signaling pathway to suppress autophagy and stimulate tumorigenesis and metastasis.

## 1 Introduction

Colorectal cancer (CRC) is a major global health problem. It is the third leading cause of cancer‐related deaths each year, with approximately 0.9 million deaths and 1.9 million new cases each year [1]. Unfortunately, CRC incidence and mortality are rapidly increasing in many countries with high and medium levels of human development, particularly in Eastern Europe, Asia, and South America [2]. The outcome of CRC is still poor owing to high metastasis and recurrence, despite significant advances in detecting and threatening CRC [3]. On account of complex pathogenesis and multiple genes, it is crucial to elucidate the molecular mechanisms involved in the development of CRC.

Methylenetetrahydrofolate dehydrogenase 1 (MTHFD1) is the enzyme with three key activities: 10‐formyltetrahydrofolate synthetase, 5,10‐methylenetetrahydrofolate cyclohydrolase, and 5,10‐methylenetetrahydrofolate dehydrogenase. They are important intermediates in one‐carbon metabolism, providing activated C1 groups for methionine, pyrimidine, and purine biosynthesis [4, 5]. Due to defective de novo thymidylate biosynthesis, mutations in MTHFD1 in humans result in severe megaloblastic anemia and combined immunodeficiency [6, 7, 8]. It has recently been reported that MTHFD1 polymorphism in humans is concerned with an increased risk of congenital heart defects and neural tube defects [9, 10]. Yu et al. detected that a high level of MTHFD1 expression on hepatocellular carcinoma tissue was independently associated with a worse prognosis [11]. The rs2236225 variant of the MTHFD1 gene might be linked to ovarian cancer [12]. Silencing MTHFD1 clearly diminished the growth and promoted the apoptosis of nonsmall cell lung cancer (NSCLC) by inhibiting DNA methylation [13]. MTHFD family genes could serve as promising prognostic markers and therapeutic targets for individuals diagnosed with bladder cancer [14]. The development of pancreatic cancer is promoted through the activation of MTHFD1 via decrotonylation at Lys354 and Lys553, leading to increased resistance to ferroptosis [15]. However, MTHFD1 expression and its role in causing and progressing CRC are not well known, which may form the basis for developing further investigation and potential new therapeutic strategies for CRC.

Autophagy is a type of homeostatic mechanism whereby the cellular package and break down cytosolic components, recycling the degradation products to sustain cellular metabolism [16, 17, 18]. Autophagy may protect against tumor formation in normal cells. However, cancer cells may become autophagic after exposure to various types of stress, such as hypoxia, radiation, and withdrawal of growth factors [19].

Several pathways are involved in regulating autophagy in different types of cancer. Autophagy can be controlled by the phosphoinositide‐3 kinase (PI3K)‐AKT‐ mammalian target of the rapamycin (mTOR) signaling pathway, which has been implicated in controlling cancer cell proliferation, differentiation, motility, and survival in many tumors [20, 21, 22, 23, 24].

The study investigated how MTHFD1 affects CRC cell line proliferation, migration, and invasion of lines in vitro, as well as the influence of MTHFD1 on CRC tumorigenesis and metastasis in mice. Furthermore, we assessed the potential function of MTHFD1 in modulating the regulation of autophagy mediated by the PI3K‐AKT‐mTOR signaling pathway, which leads to CRC cell tumorigenesis and metastasis.

## 2 Materials and Methods

### 2.1 Patient Specimens

Ninety‐one formalin‐fixed, paraffin‐embedded CRC specimens and corresponding noncancerous tissues were obtained at random from the Sixth Affiliated Hospital, Sun Yat‐sen University, Guangzhou, China. The Medical Research Ethics Committee of the Sixth Affiliated Hospital of Sun Yat‐sen University approved this study.

### 2.2 Cell Culture

CRC cell lines (SW480, HCT‐116, and DLD‐1) from American Type Culture Collection (ATCC) were cultured in Dulbecco's modified Eagle's medium (DMEM) (Gibco, Thermo Fisher Scientific, St Peters, MO, USA) containing 10% heat‐inactivated fetal bovine serum (FBS) (Gibco, Thermo Fisher Scientific, St Peters, MO, USA) and 2% penicillin/streptomycin (HyClone, Shanghai, China). All of the cell lines were grown at 37°C in a 5% CO_2_ environment. At approximately 80%–90% confluence, cells were digested by trypsinization and passaged.

### 2.3 Gene Knockdown or the Overexpression by Lentiviral Transduction

Short hairpin RNA (shRNA) in the psi‐LVRU6GP‐shRNA plasmid vector targeting MTHFD1;(psi‐LVRU6GP‐sh‐MTHFD1‐puromycin; sh‐1, 5′‐GGATCAAAGCCACTCACATTA‐3′; sh‐2, 5′‐GCCAGGAAAGTGGATGATTCA‐3′) and its control psi‐LVRU6GP‐control (sh‐NC) were obtained from Guangzhou FulenGen Co. Ltd., China. Lentiviral constructs for overexpression MTHFD1(pLenti‐CMV‐MTHFD1‐puromycin) were obtained from Geneppl Technology, Co. Ltd., China. Transfection of HCT‐116, DLD‐1, and SW480 cells was performed with Lipofectamine 3000 (Invitrogen Preservation, Carlsbad, CA, USA) in accordance with the manufacturer's protocol. After an incubation period of 24 h, the medium has been removed and replaced with the same amount of medium. At the end of the 48‐h incubation period, the cell cultures were treated with appropriate concentrations of puromycin to eliminate cells with failed viral transduction. Western blot (WB) was performed for the evaluation of the knockdown and overexpression efficiency.

### 2.4 RNA Extraction and Reverse Transcription‐Quantitative Polymerase Chain Reaction (RT‐qPCR)

TRIzol reagent (Thermo Fisher Scientific, St Peters, MO, USA) was used to obtain total RNA from cells or tissue samples. A NanoDrop ND‐2000 spectrophotometer (Thermo Fisher Scientific, St Peters, MO, USA) was subsequently determined to assess RNA concentrations and purity. The ReverTra Ace qPCR RT kit (Toyobo Biochemicals, Kita‐ku, Osaka, Japan) was used to conduct reverse transcription according to the manufacturer's protocol. Quantitative real‐time reverse transcription PCR (qRT‐PCR) was conducted on an Applied Biosystems 7500 Sequence Detection system with the SYBR Green PCR Master Mix (Applied Biosystems, Foster City, CA, USA). The internal reference was GAPDH. The expression of genes' relative mRNA was calculated by using the 2^−ΔΔ*C*q^ method.

### 2.5 Immunohistochemistry (IHC)

Formalin was used to fix tissue samples of CRC, which were embedded in paraffin. Slides were then prepared by coating them with either an anti‐MTHFD1 rabbit polyclonal antibody (1:200 dilution; Abcam, USA) or an anti‐Ki‐67 rabbit monoclonal antibody (1:200 dilution; Beyotime, China), which was incubated 14–16 h at 4°C, flushed with PBST. The slides were treated with the corresponding amount of enzyme‐marked goat anti‐mouse/rabbit IgG polymer (ZSGB‐BIO, China) for 30 min at a temperature of 37°C and subsequently flushed with PBST. Furthermore, the peroxidase reaction was performed by using 3,3′‐diaminobenzidine (DAB) (ZSGB‐BIO, China). Tissue sections were subjected to hematoxylin staining. The scoring system for IHC intensity was 0 to 4 points. Cells were classified into five groups to determine based on the percentage of positive cells: more than 75% of positive cells, 50%–75% of positive cells, 25%–50% of positive cells, less than 25% of positive cells, and no positive.

### 2.6 Cell Proliferation Assay

A Cell Counting Kit‐8 (CCK8) (Biosharp, China) was utilized to measure cellular growth. In brief, stably transfected SW480, DLD‐1, and HCT‐116 cell suspension (100 μL/well) were inoculated into a 96‐well plate. Each well was seeded with 1000 cells for the cell proliferation assay and incubated in an environment of 5% CO_2_ at 37°C for 2 h. A microplate reader (Thermo Fisher, USA) was utilized to detect the absorbance of each well at 450 nm. CCK‐8 reagents were added at 1, 2, 3, 4, and 5 days.

Colony formation assays were also used to determine the ability of the cells to proliferate. CRC cells were cultured for a period of 14 days in 6‐well plates at 500 cells per well. Immobilization of cells was performed with 4% paraformaldehyde for 10 min, followed by staining with 1% crystal violet for 15 min and rinsing with PBS.

### 2.7 Cell Migration and Invasion Assays

Transwell chambers (Corning, USA) were utilized to evaluate the ability to migrate and invade. Stably‐transfected cell lines (SW480, DLD‐1, and HCT‐116) were digested with trypsinization and suspended in DMEM. For migration, serum‐free medium in a 24‐well plate was initially seeded in the upper chamber with 1 × 10^5^ cells (200 μL). In the lower chamber, 700 μL of medium supplemented with 10% FBS was added. Cells were grown at 37°C for 48 h. Immobilization of migrated cells was performed with 4% paraformaldehyde for 15 min, followed by staining with 0.1% crystal violet for 30 min and microscopically counted.

For the migration test, the Matrigel (BD Biosciences, USA) was mixed 1:8 with the DMEM to prepare a working concentration. Fifty microliters of the mixture were applied on the top of the Transwell chamber of the 24‐well plate at 37°C for 4 h. The upper chamber was seeded with a suspension of 2 × 105 cells (200 μL) with serum‐free medium. As with the migration experiment, the subsequent steps were identical.

### 2.8 Transmission Electron Microscopy (TEM)

To prepare the cells for imaging by TEM, they were subjected to a series of procedures. The cells were first washed, then digested and centrifuged to collect the cells. They were then fixed with a TEM fixative (Servicebio, Wuhan, China). Different concentrations of acetone were used to desiccate the fixture of the stably transfected cells with Epon 812 resin. Specimens were then sectioned at 60–80 nm, followed by staining with sodium acetate and lead citrate. Finally, cells were visualized by transmission electron microscopy (HT7700; HITACHI, Tokyo, Japan).

### 2.9 WB Analysis

The BCA protein assay kit (Sigma‐Aldrich, USA) was utilized to extract the total protein of the CRC cell lines. RIPA Lysis Buffer was then used to lyse the cell protein. Protein was separated through sodium dodecyl sulfate‐polyacrylamide gel electrophoresis (SDS‐PAGE) and then electrotransferred to polyvinylidene fluoride membranes (PVDF) (Millipore, USA). To prepare membranes for antibody detection, they were blocked for 2 hour in 5% skimmed milk PBST and subsequently incubated for 14 h with the following antibodies at 4°C. After incubation and washing with PBST for 15 min, membranes were then incubated for 1 hour at 37°C with goat anti‐rabbit/mouse antibodies, followed by another wash with PBST. The membranes were imaged by the Bio‐Rad gel imaging system. Image J software (version 6.0, USA) was used for measuring band quantification.

### 2.10 In Vivo Experiments

BALB/c mice, 4 weeks of age, were purchased from Gempharmatech (Guangdong, China). They have been in special pathogen‐free housing conditions. Animal experiments were conducted with the permission of the Institutional Animal Care and Use Committee of the Sixth Affiliated Hospital, Sun Yat‐sen University, Guangzhou, China (No. IACUC 2022021102). All mice were monitored by dedicated caregivers and given access to water and food freely. Mice were randomized to control and experimental groups (HCT‐116‐sh‐NC and HCT‐116‐sh‐MTHFD1). The volume of tumors was assessed every 3 days with a caliper and was formulated as *V* (mm^3^) = length × width^2^ × 0.5. At the termination of the study, the mice were anesthetized, euthanized, and disposed of in a humane manner. Samples of tumors were harvested and prepared for analysis.

### 2.11 Statistical Analysis

Data, presented as mean ± SD, were analyzed using GraphPad Prism 9.0 software. A student's *t*‐test was used to analyze continuous variables. Data were also analyzed using SPSS 17.0 (SPSS Inc., Cary, North Carolina, USA). A value of *p* < 0.05 was regarded as statistically significant.

## 3 Results

### 3.1 MTHFD1 Is Significantly Overexpressed in CRC Patients

**FIGURE 1:**
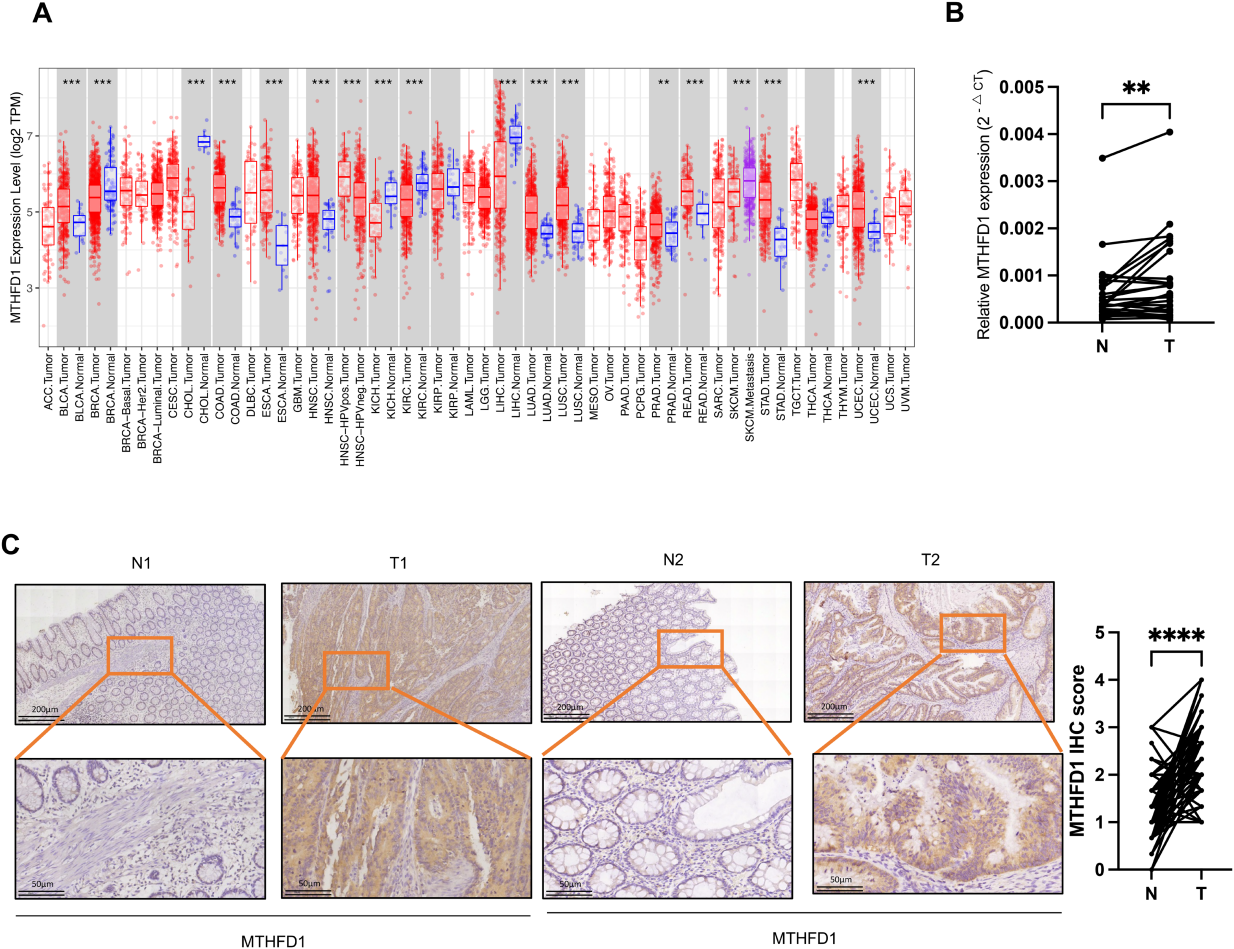
MTHFD1 expression levels are higher in CRC tissue than in the adjacent normality tissue. (A) the levels of MTHFD1 mRNA were analyzed by the TIMER database in different tumors and normal tissues. (B) MTHFD1 mRNA expression levels were determined in 36 samples of CRC and corresponding noncancerous tissues by RT‐qPCR. (C) In 91 CRC tissues and corresponding noncancerous tissues, immunohistochemical staining, and detection of MTHFD1 protein were performed. Data are presented as mean ± SD; *p < 0.05, **p < 0.01, ***p < 0.001, and ****p < 0.0001.

To compare how MTHFD1 expression differs between tumor and normal tissues, the TIMER database was used to investigate MTHFD1 mRNA levels in tumors and corresponding normal tissues from multiple cancer types (Figure 1A). This investigation revealed that the MTHFD1 mRNA levels were distinctly elevated in the bladder, colorectal, esophageal, head, and neck, lung, prostate, gastric, and endometrial cancers compared to the normal tissues. In our patient cohort, the matched CRC samples and normal adjacent frozen tissues validated this observation by using qPCR and IHC (Figure 1B,C). Based on the results, we speculated that highly expressed MTHFD1 may have a significant role in modulating the function of CRC cells.

### 3.2 MTHFD1 Promotes the Proliferation of CRC Cells In Vitro

**FIGURE 2:**
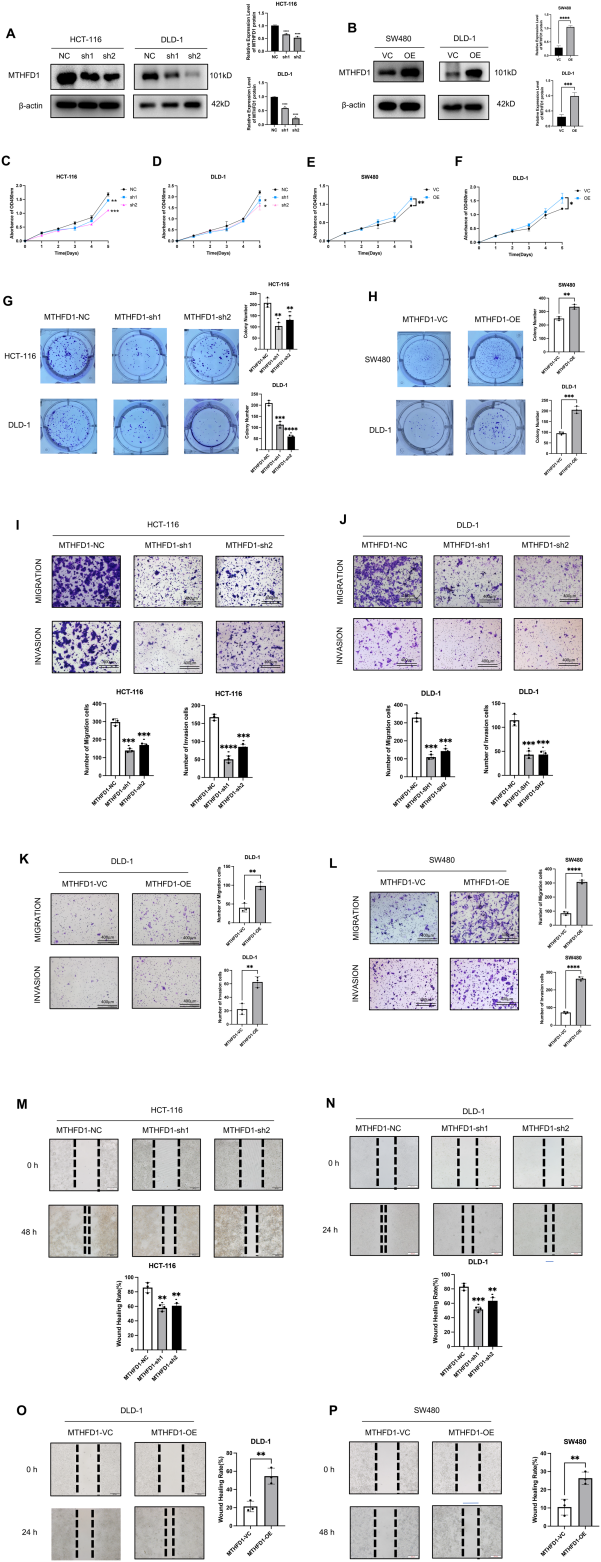
MTHFD1 knockdown inhibits CRC cell proliferation, migration, and invasion. (A) The efficiency of MTHFD1 knockdown in HCT‐116 and DLD‐1 was accessed by WB. (B) The efficiency of MTHFD1 overexpression in SW480 and DLD‐1 was detected by WB. (C–F) CCK‐8 assays were used to assess cellular proliferation. (G, H) Colony formation assays detected cell proliferation. (I–L) MTHFD1 knockdown was shown to reduce cell migration and invasion in Transwell assays. (M–P) Cell migration abilities were measured by the wound healing assays. All experiments in vitro were carried out in triplicate and were repeated a minimum of three times. Data are presented as mean ± SD; *p < 0.05, **p < 0.01, ***p < 0.001, and ****p < 0.0001.

In order to determine MTHFD1's roles in CRC, we initially examined the relative MTHFD1 mRNA in CRC cell lines. Because of the moderate levels of MTHFD1 mRNA, HCT‐116, DLD‐1, and SW480 cells were chosen to perform the following experience. To test this hypothesis, stable MTHFD1 overexpression cells were established in DLD‐1 and SW480, and knockdown cells in DLD‐1 and HCT‐116 cells. WB was performed to verify MTHFD1 protein expression levels, and these cell lines were used for subsequent assays (Figure 2A,B). DLD‐1 and SW480 cells overexpressing MTHFD‐1 were more proliferative than control cells, as determined by the Cell Counting Kit‐8 (CCK‐8) assay (Figure 2C,D). On the contrary, the knockdown of MTHFD1 in HCT‐116 and DLD‐1 cells showed decreased proliferative capacity (Figure 2E,F). Meanwhile, HCT‐116 and DLD‐1 cell proliferation was suppressed by low expression of MTHFD1 in colony formation assays (Figure 2G,H). Taken together, these observations demonstrate that overexpressing MTHFD1 accelerates CRC cell proliferation.

### 3.3 MTHFD1 Enhances Migration and Invasion of CRC In Vitro

To investigate how MTHFD1 affects cancer progression and metastasis, we evaluated its effect on cell migration and invasion potential using the wound healing assay and the Transwell assay, which demonstrated that MTHFD1 overexpression enhanced the invasion and migration capabilities of DLD‐1 and SW480 cells (Figure 2I–P). On the contrary, MTHFD1 knockdown caused a noticeable decrease in the ability of DLD‐1 and HCT‐116 cells to migrate and invade.

### 3.4 MTHFD1 Knockdown Suppresses CRC Proliferation and Metastasis In Vivo

**FIGURE 3:**
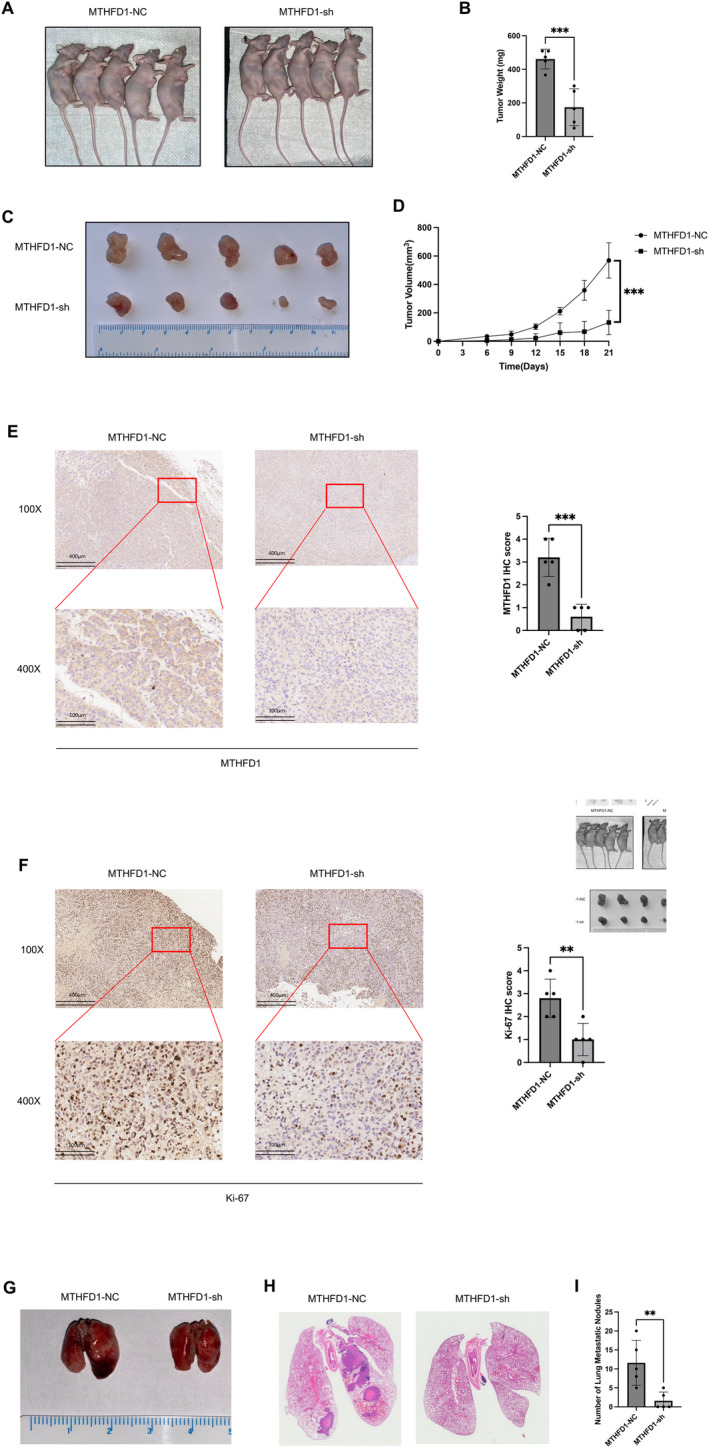
MTHFD1 knockdown modulates tumorigenesis proliferation and metastasis in vivo. (A) Photographs of nude mice were captured 21 days postinjection with either control cells or cells with MTHFD1 knockdown. (B–D) After sacrificing the mice, the tumors were weighed and the volume and growth curve of the tumors were measured. (E, F) The expression levels of MTHFD1 and Ki‐67 proteins of the tumors harvested from the nude mice were measured. (G) Photographs of the lungs of nude mice were taken 2 months postinjection via the lateral tail vein with either control cells or cells with MTHFD1 knockdown. (H, I) H&E staining was performed to visualize metastatic tumor nodules in the lungs of nude mice. Data are presented as mean ± SD; *p < 0.05, **p < 0.01, ***p < 0.001, and ****p < 0.0001.

To determine whether down‐regulation of MTHFD1 in CRC cells could inhibit tumorigenesis and metastasis in vivo, nude mice received HCT‐116 cells stably transfected with the knockdown of MTHFD1 or the control groups by injection. The tumors with the knockdown of MTHFD1 grew much slower and had a smaller average volume than those of the controls (Figure 3C,D). Mice were euthanized 21 days after injection (Figure 3A). The average tumor weights of the knockdown of MTHFD1 were 450 mg, and it was 178 mg in controls (Figure 3B). In addition, protein expression of MTHFD1 and Ki‐67 in pathological sections was assessed by immunohistochemical analysis (Figure 3E,F). The results showed that the MTHFD1 and Ki‐67 protein expressions were reduced in comparison to controls. In further investigation of the impact of MTHFD1 on metastasis, nude mice were treated by injection of HCT‐116 cells stably transfected with MTHFD1 knockdown or controls into the lateral tail vein. After 2 months, the mice were euthanized, and the lung metastasis was analyzed. Hematoxylin and eosin (H&E) staining analysis revealed a lower rate of lung metastasis and fewer tumor metastatic nodules in nude mice with MTHFD1 knockdown compared to the control group (Figure 3G–I). These findings from in vitro cell assays and in vivo experimentations suggest MTHFD1 has been implicated in CRC progression and metastasis.

### 3.5 MTHFD1 Inhibits Autophagy of CRC Cells

**FIGURE 4:**
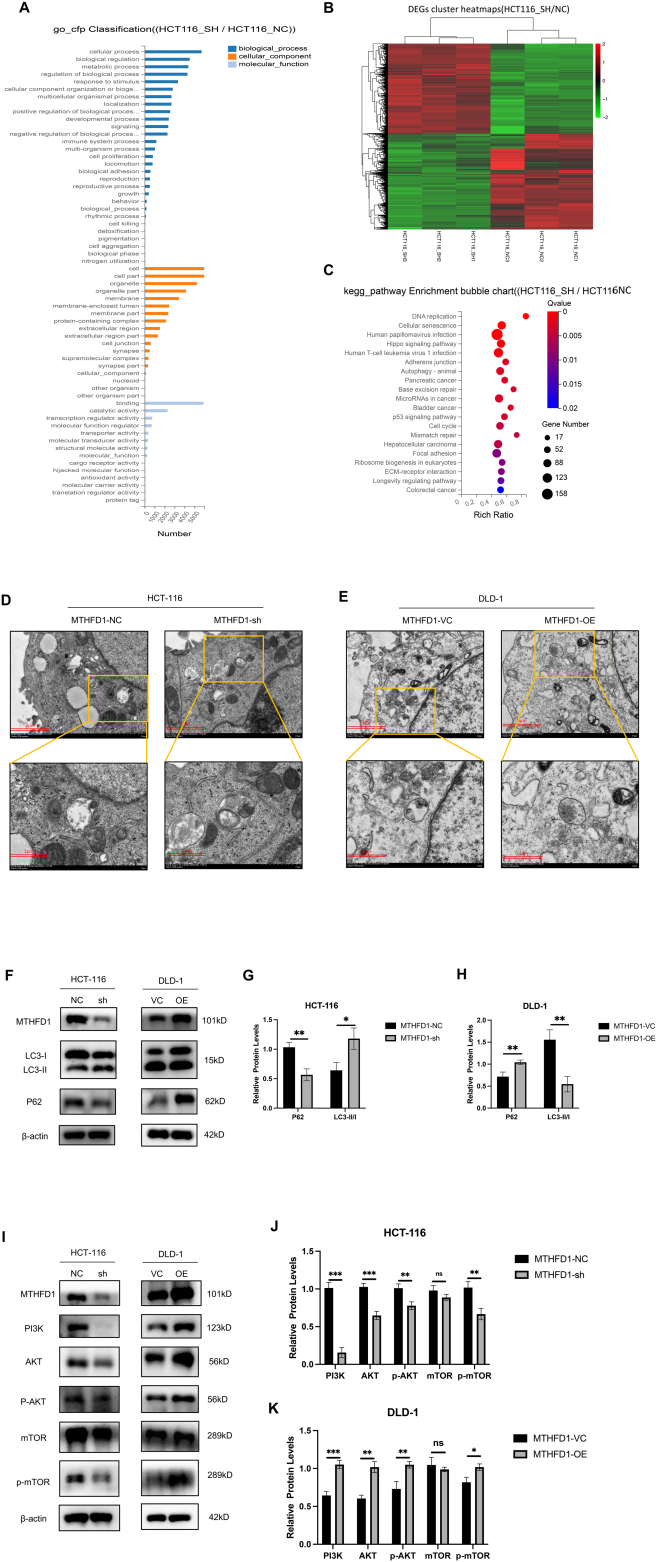
Low MTHFD1 expression can enhance autophagy in CRC cells via the PI3K‐AKT‐mTOR signaling pathway. (A–C) Analysis of the differentially expressed genes associated with the expression of MTHFD1 using GO and KEGG pathways. (D, E) The autophagosomes of HCT‐116 and DLD‐1 cells were observed by TEM. (F–H) The measurement of autophagy‐associated proteins P62, LC3, Beclin1, and ULK1 in CRC cells was performed using WB. (I–K) WB examined relevant PI3K‐AKT‐mTOR signaling pathway protein levels in CRC cells. All experiments in vitro were carried out in triplicate and were repeated at least three times. Data are presented as mean ± SD; *p < 0.05, **p < 0.01, ***p < 0.001, and ****p < 0.0001.

Further investigation into the mechanism of action of MTHFD1, we performed RNA‐Seq on HCT‐116 cells with MTHFD1 knockdown and the control cells. Our analysis identified 6541 different expressed genes (DEGs) that contained 3160 upregulated and 3381 downregulated genes. Meanwhile, the 3851 target genes were subjected to GO and KEGG pathway enrichment analysis, showing that autophagy was significantly enriched (Figure 4A–C). To validate this finding, we conducted TEM and WB experiments to assess the effect of MTHFD1 on autophagy. TEM revealed that the knockdown of MTHFD1 significantly induced autophagosome formation in HCT116 cells, while overexpression of MTHFD1 expression in DLD‐1 cells resulted in a decrease in autophagosome formation (Figure 4D,E). To further understand the autophagy‐modulating effect of MTHFD1 in CRC cells, the expression of well‐known autophagy‐related proteins (Beclin1, LC3II/I ratio, p62, and ULK1) was detected by WB [25]. Additionally, WB showed that overexpressing MTHFD1 in SW480 cells markedly decreased the Beclin1 and ULK1 protein expression and the ratio of LC3‐II to LC3‐I and elevated P62 expression, indicating that MTHFD1 inhibited autophagy in CRC cells. Whereas MTHFD1 knockdown in HCT‐116 cells promoted Beclin1, ULK1 protein expression and the ratio of LC3‐II to LC3‐I, and down‐regulated P62 expression (Figure 4F–H), indicating that knockdown of MTHFD1 induced autophagy in CRC cells. Overall, these findings suggest that MTHFD1 overexpression suppresses autophagy induction whereas MTHFD1 inhibition promotes autophagy in CRC cells.

### 3.6 MTHFD1 Regulates the PI3K‐Akt‐mTOR Pathway

It has been reported that the PI3K‐AKT‐mTOR signaling pathway can regulate autophagy in cancer cells [26]. Based on the above‐mentioned, we hypothesize that MTHFD1 plays an important function in determining the tumorigenic properties of CRC cells through autophagy and that this effect partly involves the PI3K‐AKT‐mTOR signaling pathway. Based on this hypothesis, we aimed to quantify the protein expression levels of key pathway components in MTHFD1‐ overexpressing SW480 cells and MTHFD1‐knockdown HCT‐116 cells by using WB (Figure 4I–K). The findings showed that PI3K expression was severely depressed in MTHFD1 knockdown HCT‐116 cells, and MTHFD1 overexpression in SW480 cells elevated PI3K expression. In addition, the MTHFD1‐overexpressing SW480 cells exhibited higher levels of phospho‐Akt (p‐Akt), and phospho‐mTOR (p‐mTOR) expression compared to the corresponding control cells. Conversely, MTHFD1 knockdown in HCT‐116 led to decreased expression of p‐mTOR. The results demonstrated that MTHFD1 acts as a regulator of the PI3K‐Akt‐mTOR signaling pathway.

### 3.7 MTHFD1 Promotes CRC Cells to Proliferate, Migrate and Invade via Autophagy Mediated

**FIGURE 5:**
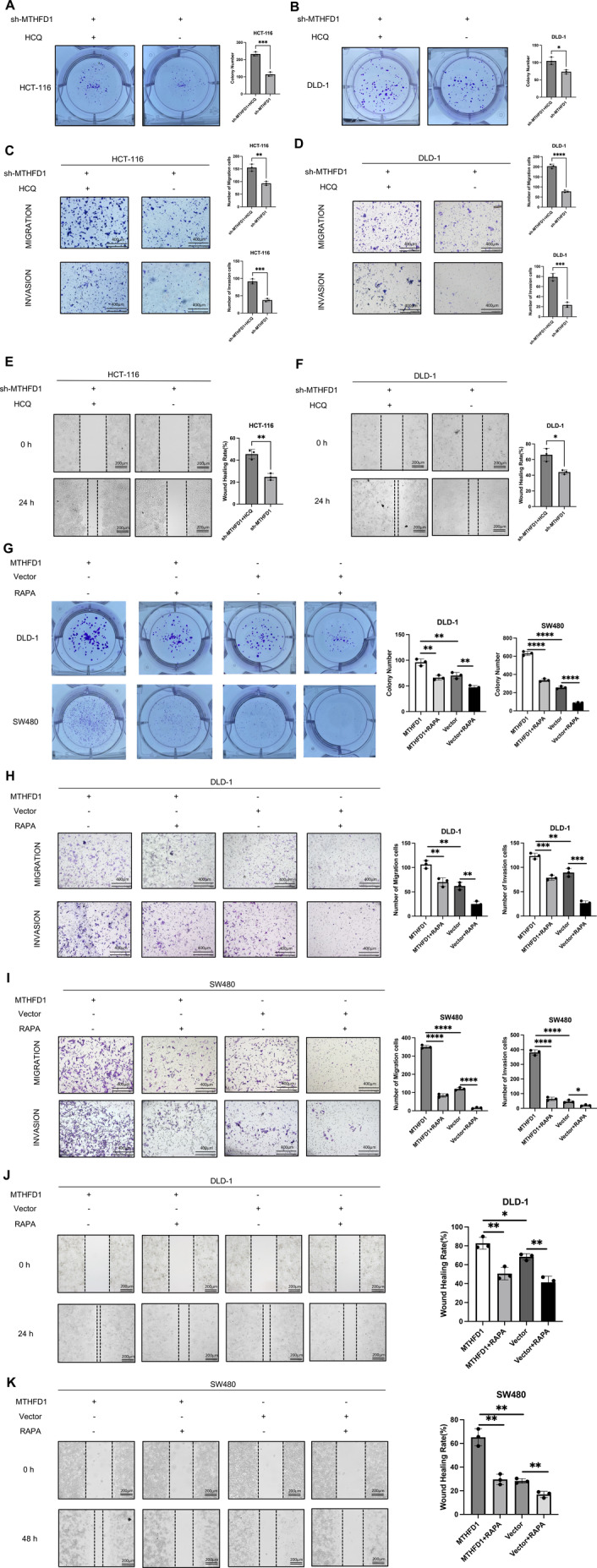
MTHFD1 promotes the proliferation, migration, and invasion of CRC cells through the mediation of autophagy. (A, B) The colony assays showed that HCQ enhanced the proliferation potential. (C–F) The Transwell assays and wounding assays showed that HCQ enhanced the migration and invasion potential. (G) The colony assays showed that RAPA suppressed the proliferation of MTHFD1 overexpression cells. (H–K) The Transwell assays and wounding assays showed that RAPA inhibited the migration and invasion of MTHFD1 overexpression cells. All experiments in vitro were carried out in triplicate and were repeated at least three times. Data are presented as mean ± SD; *p < 0.05, **p < 0.01, ***p < 0.001, and ****p < 0.0001.

The anti‐proliferation and anti‐metastasis effects of MTHFD1 suppression were reversed when MTHFD1‐knockdown DLD‐1 and HCT‐116 cells were cotreated with Hydroxychloroquine (HCQ), an autophagy inhibitor (Figure 5A–F). Whereas the Transwell assays and the wounding assays revealed that MTHFD1‐ overexpressing DLD‐1 and SW480 cells that were treated with the autophagy activator Rapamycin (RAPA) exhibited worse cell migration and invasion capabilities (Figure 5H–K). Meanwhile, colony‐forming experiments demonstrated that cell proliferation ability was inhibited in the MTHFD1‐overexpressing DLD‐1 and SW480 cells that were treated with the autophagy activator RAPA (Figure 5G). Therefore, overexpressing MTHFD1 enhanced CRC cells to proliferate, migrate, and invade through inhibiting autophagy. However, suppression of MTHFD1 inhibited the migration and invasion ability of CRC cells through the induction of autophagy.

## 4 Discussion

To compare differences in MTHFD1 expression in public databases and in our own cohort of patients, which showed that we identified significantly increased expression of MTHFD1 in CRC tissue compared to nearby nontumorous tissue. Results from in vivo and in vitro experimentation indicated that MTHFD1 induced CRC cells to proliferate, invade, and migrate. Mechanistically, we discovered that MTHFD1 knockdown induces autophagy, resulting in the promotion of CRC cells to proliferate, invade, and migrate, partly by inhibition of the PI3K‐AKT‐mTOR signaling pathway. Conversely, MTHFD1 overexpression may promote PI3K expression levels and enhance the expression levels of p‐AKT and p‐mTOR, leading to the inhibition of autophagy and promotion of CRC cells to proliferate, invade, and migrate.

Autophagy is a lysosomal degrading pathway. It has an adaptive role in protecting organisms from many harmful conditions [27]. Compared with noncancerous cells, cancer cells have a great reliance on autophagy to limit cell damage and maintain viability under adverse conditions in the face of more environmental and intrinsic metabolic stressors [28]. Inhibition or activation of autophagy has recently been shown to be important in tumor growth [29, 30]. Inhibition of autophagy dampened the activation of the Wnt/β‐catenin signaling pathway [31]. The increased nuclear accumulation of β‐catenin contributes to heightened resistance to chemotherapy and radiotherapy in advanced rectal cancer [32]. In the meantime, loss of autophagy was found to be an important prerequisite for the development of cancer [33]. We found that MTHFD1 knockdown increased LC3‐I to LC3‐II conversion and decreased P62 expression. The autophagy of CRC cells may be facilitated by the low expression of MTHFD1. Thus, autophagy could regulate cellular proliferation, invasiveness, and metastasis under conditions of stress. Hence, we examined the association between autophagy and CRC through CRC cells co‐treated with HCQ, an autophagy inhibitor, or RAPA, an autophagy activator. The analysis demonstrated that HCQ increased the proliferation, invasiveness, and metastasis of CRC cells with low levels of MTHFD1, and RAPA reduced those with high levels of MTHFD1. Hence, overexpressing MTHFD1 in CRC cells increased proliferation invasion and migration partly through the inhibition of autophagy.

Earlier studies have implicated the PI3K‐AKT‐mTOR signaling pathway in multiple human pathological conditions, such as proliferative disorders and carcinoma [26, 34, 35, 36]. Moreover, a regulator of autophagy in cancer cells may be the PI3K‐AKT‐mTOR pathway [37]. As a result, targeting the PI3K pathway is now considered a highly effective therapeutic strategy for these ailments and is a major focus of drug development. The PI3K‐AKT‐mTOR signaling pathway has been suggested to be the main pathway that is involved in initiating and regulating autophagy [38, 39, 40]. Several studies have observed activating AKT‐inhibited autophagy and inhibiting AKT‐induced autophagy [41, 42]. Inhibiting the PI3K‐AKT‐mTOR pathway promotes cellular autophagy. The autophagic pathway may be regulated by downstream sites of mTOR, which is the key node in the PI3K‐AKT‐mTOR pathway. This study showed that overexpressing MTHFD1 partly increased PI3K, p‐AKT, and p‐mTOR expression to inhibit autophagy and increase CRC cell proliferation invasion and migration. Indeed, one of the common mechanisms of tumorigenesis and progression is an imbalance in the PI3K‐AKT‐mTOR signaling pathway [43]. Thus, this study provides a novel mechanism for the role of MTHFD1 in CRC by regulating the PI3K‐AKTmTOR signaling pathway. At the same time, this study has certain limitations. The specific mechanism of high expression of MTHFD1 is unclear and needs further exploration and verification. Additionally, the therapeutic effect of MTHFD1 inhibitors on CRC PDX or organoids is warranted in our future work to further clarify the clinical value of targeting MTHFD1.

To summarize, we show that MTHFD1 is upregulated in patients with CRC. Functionally, we identified a novel role for MTHFD1 in modulating PI3K‐Akt‐mTOR‐induced autophagy as well as subsequent biological processes such as cell proliferation, migration, and invasion. Targeting both MTHFD1 and the PI3K‐AKT‐mTOR signaling pathway concurrently represents a promising strategy for inhibiting tumor growth and metastasis.

## Author Contributions

**Zhihao Li:** conceptualization (equal), data curation (equal), formal analysis (equal), methodology (equal), writing – original draft (equal). **Haoxian Ke:** methodology (equal). **Jiawei Cai:** methodology (equal). **Shubiao Ye:** conceptualization (equal), writing – review and editing (equal). **Junfeng Huang:** methodology (equal). **Chi Zhang:** methodology (equal). **Ming Yuan:** methodology (equal). **Ping Lan:** conceptualization (equal), funding acquisition (equal), project administration (equal), supervision (equal), writing – review and editing (equal). **Xianrui Wu:** conceptualization (equal), project administration (equal), supervision (equal), writing – review and editing (equal).

## Ethics Statement

The study was conducted in accordance with the Declaration of Helsinki, and approved by the Medical Research Ethics Committee of the Sixth Affiliated Hospital, Sun Yat‐sen University.

## Conflicts of Interest

The authors declare no conflicts of interest.

## Data Availability

The data that support the findings of this study are available from the corresponding author upon reasonable request.
